# 

**Published:** 1918-06-29

**Authors:** 


Rates of Subscription: Twelve months, 6/6; Six months, 4/-; three months, 2/-, post free to any address in the United Kingdom. Abroad, Twelve months, 8/8; Six months, 5/-; Three months, 2/6, post free. THE HOSPITAL "Index" to Volume LXI, October 1917 to March 1918, is now ready, price 3½d. per copy, post free. Address The Manager, The Hospital, "The Hospital" Building, 28/29 Southampton Street, Strand, London, W.C. 2.

To obtain **The HOSPITAL**
regularly **EVERY THURSDAY** a definite order **must** be placed with your Newsagent **at once.**
All Newsagents, Booksellers, and Railway Bookstalls will gladly book your order, but copies cannot be stocked for sale to chance customers.

### The Too Officious Visitor.

According to the Kendal Health Committee's report, a complaint by the local doctors of undue activity on the part of the health visitor was presented. The Committee decided that the complaint should be investigated and a communication addressed to the doctors. The report does not reveal the details.

To obtain **The HOSPITAL** regularly **EVERY THURSDAY** a definite order **must** be placed with your Newsagent **at once.** All Newsagents, Booksellers, and Railway Bookstalls will gladly book your order, but copies cannot be stocked for sale to chance customers.

### HOSPITAL AND INSTITUTIONAL RESULTS.

**Poplar Hospital for Accidents.**—Year after year we turn to the accounts of this hospital to see if the famous boast of "never in debt" is still justified, and each time we rejoice to find that it still holds good. In 1917 the hospital excelled itself and had a balance to the good of £8,000, of which £6,000 has been allocated to a suspense account for proposed buildings, alterations, and repairs.

**Royal Infirmary, Sheffield.**—The one hundred and twentieth annual report is reduced, in the interests of economy, to the barest limits. The cost per bed per day on total ordinary expenditure was 6s. The work-people of Sheffield contributed in 1917 £1,210 in excess of the amount subscribed in 1916. The total expenditure was increased by £4,600, and was £6,200 in excess of income. Sheffield, as a city, is at present in a position to put this right, and should make it a point of honour to do so.

**Royal London Ophthalmic Hospital.**—On an average 358 out-patients attend daily, in spite of a system of limitation necessitated by the shortage of surgeons. After a run of bad luck in a financial sense the hospital has turned the corner, and the accounts showed a balance of £2,982 to the good last year.

**Mount Vernon Hospital**—The report, as usual, is tasteful in its get-up and well arranged. The plan of concentration of the in-patient work at Northwood has been successful, and the institution continues to prosper.

### Windfalls of Whisky.

SOME hospitals often benefit in mysterious ways. The V.A.D. Hospital at Mill Dam, South Shields, has just secured a bottle of port and one bottle and two flasks of whisky. These good gifts, says the Chief Constable, "came into the possession of the police," and were not claimed, and so he handed them over to the hospital for the benefit of the patients. The police, naturally, would have no use for such things!

### Gifts and Bequests.

The Royal Dental Hospital, Leicester Square, W.C., has received a special donation of £250 from the Worshipful Company of Goldsmiths, a donation of five guineas from the Trustees of Berman's Charity, a donation of £50 from the Worshipful Company of Clothworkers, and one of £100 from the Worshipful Company of Grocers.

Lieutenant Samuel Worthington, R.H.A., a barrister, who was killed in Palestine on November 28, left £500 to the Metropolitan Sunday Fund.

Mr. Humphrey Roberts, J.P., of Pendleton, left £5,000 to the Salford Royal Hospital for the endowment of two beds, and any balance for general purposes.

Under the will of Miss J. E. Hilton, of Manchester, £200 was left to the Missions to Seamen, £300 to the Manchester Sick Poor and Private Nursing Institution, and £500 each to the Ancoats Hospital and Dispensary and St. Mary's Hospital for Women and Children, Manchester.

The Board of Management of the Manchester Royal Infirmary has received a donation of £125 from Nurse Jones, of the infirmary staff, in memory of the "Royal Manchesters" and in honour of her father's birthday.

Amongst the bequests in the will of the late Mr. W. W. Howard, of Mill Hill, who left estate valued at £128,833, is one of £20,000 for the benefit of wounded soldiers. The residue of the estate is to be given to such descendants of his parents, his friends, and such charitable institutions as his brother Walter shall determine.

Nottingham General Hospital, the General Dispensary, the Hospital for Children, the Eye Hospital, and the Women's Hospital have each been left £100 under the will of Mr. John Berrey, of Nottingham.

A number of legacies have been left under the will of the late Mr. William Paulden, of Wilmslow, including £1,000 to Manchester Royal Infirmary, £500 each to St. Mary's Hospital, Manchester, and the Mauldeth Hospital for Incurables, and £250 each to Hulme Dispensary and Chorlton-on-Medlock Dispensary.

Sir Reuben Barrow, of Croydon, has left £2,000 to the Croydon General Hospital and £500 to the Reedham Orphanage.

The residue of the estate of Brigade-Surgeon John Law, M.D., who left £49,953, is bequeathed to King Edward's Hospital Fund; amongst the specific legacies are £1,000 each to the Seamen's Hospital, Greenwich, the Devon and Exeter Hospital, and the Royal Infirmary, Edinburgh.

In the will of Dr. Henry Maudsley it is stated that a promise had been made to pay £10,000 to the London County Council for the establishment of the "Maudsley Hospital," and it is directed that the trustees should pay this sum if it has not already been paid.

Alderman R. D. and Mrs. Ward, of Halifax, have given £1,000 to the Royal Infirmary for the endowment of a bed in celebration of their golden wedding.

The Chelsea Football and Athletic Co., Ltd., have sent a contribution of twenty-five guineas in response to the Lord Mayor's appeal on behalf of the Royal Hospital and Home for Incurables, Putney Heath.

Mr. David George, of Normanton Road, South Croydon, manager of the Bank of New South Wales, Threadneedle Street, E.C., bequeathed, on the death of his wife and his daughter and her husband, £2,500 to the Bignold Cottage Hospital, Wick, and £1,000 to the Croydon General Hospital.

### Passing Events and Topics.

### Removed from the Register.

The London County Council has removed from the Register of War Charities the charities known as the Great Britain to Poland Fund and the Russian Flag Day.

### Bottles for the Italian Red Cross.

The Italian Red Cross is making active appeal to the Milanese citizens to supply empty bottles of all sorts and kinds, which are to be refilled gratuitously with mineral waters through the generosity of various Italian mineral-water companies.

### The Woman Doctor in Melbourne.

Dr. Isabella Phillips, M.B. and Ch.B. (Melb.), has been registrar of the Melbourne Hospital for the last year, and has also served lately as acting superintendent during the absence on holiday of Dr. McMeekin. This is the first time in the history of the institution that a woman has filled either of these positions.

### A Lost Medal Replaced.

At a meeting at the Royal Devon and Exeter Hospital, when medals were presented to nurses heading the list in a recent examination, it was announced that one recipient of a silver medal (Nurse Sawtell) was on a torpedoed ship and lost her medal, but the committee had decided to give her another in place of it.

### Red Cross Shopkeeping Enterprise.

The annual report of Red Cross work in Gloucestershire, always an engaging and well-presented document, this year, probably for reasons of economy, contains no illustrations. The reading matter is, however, unusually interesting, especially that part which deals with food difficulties—a subject one tries to get away from, but which has a curious and irresistible fascination. The County Director, like most people, experienced a shortage amounting to an actual difficulty, and courageously decided to set up as a wholesaler himself. A depôt was set up in Gloucester, in a shop kindly lent by a sympathiser, where such necessaries as sugar, syrup, tea, cocoa, and margarine are stored and distributed to the many hospitals in the county. Goods are purchased wholesale and sold at cost price to hospitals. Commandants requisition for what they require once a month, and issues are made to them in the first few days of the month, the County Director satisfying himself that rations, especially of sugar, have not been exceeded. A lucky purchase of sultanas resulted in the Gloucestershire Christmas puddings turning out above the average. Altogether the scheme has worked admirably, and we should not be surprised to learn that it has been adopted also in other counties.

### Infection.

A baby smiled in its mother's face;
The mother caught it, and gave it then
To the baby's father—serious case—
Who carried it out to the other men;
And every one of them went straight away,
Scattering sunshine thro' the day.

*The Modern Hospital.*

### Matrons' and Sisters' Appointments.

INFANTS' HOSPITAL, WESTMINSTER.—Miss Dora Mary Marshall has been appointed out-patient and research sister. She was trained at Westminster Hospital and at the National Hospital, Queen Square.

LEIGH JOINT FEVER HOSPITAL, ASTLEY, NEAR MANCHESTER.—Miss Maggie Milner has been appointed sister. She was trained at the Salford Union Hospital, where she was later staff nurse and sister; she has been sister at the Eastern Hospital, Homerton, and sister superintendent of St. George's Home, Chelsea.

LONDON HOMŒOPATHIC HOSPITAL, W.C.—Miss Eva Mary Stringer has been appointed out-patient sister at this institution, where she trained and has been night sister.

NATIONAL HOSPITAL FOR DISEASES OF THE HEART, LONDON.—Miss Cecilia Beaton has been appointed acting matron. She was trained at the Taunton and Somerset Hospital, has been ward sister at the Worcester General Infirmary and at the County Hospital, Bedford, and home sister at the Bolingbroke Hospital, Wandsworth Common.

NORTHAMPTON GENERAL HOSPITAL.—Miss Annie Askew has been appointed sister. She was trained at the Parish of Portsmouth Workhouse Infirmary, has been staff nurse at the Royal London Ophthalmic Hospital, and sister at the Birmingham and Midland Eye Hospital.

NORWICH CITY ASYLUM, HELLESDON, NORWICH.—Miss E. A. Cleary has been appointed matron. She was trained at the Prince of Wales' Hospital, London, and Gartlock Mental Hospital, has been sister in the Concentrated Camps, South Africa, and at the Eastern Fever Hospital, London, night superintendent at the District Asylum, Melrose, and assistant matron at the Royal Asylum, Edinburgh, and at the Stirling District Asylum, Larbert.

THE SANATORIUM, MIDDLESBROUGH.—Miss Agna Lilley has been appointed sister. She was trained at the Borough Hospital, South Shields, has been staff nurse at the Dean's Hospital, South Shields, and night charge nurse at the West Lane Hospital, Middlesbrough.

TOWNLEY'S MILITARY HOSPITAL, BOLTON.—Miss E. Fletcher has been appointed home sister. She was trained at Mill Road Infirmary, Liverpool, and in midwifery at Walton Infirmary, Liverpool, where she was staff nurse. She has been ward sister at the City of Westminster Infirmary, Hendon; ward sister and night sister at the Maternity Hospital, Birmingham; and night superintendent at the City Hospital, Newcastle-on-Tyne.

V.A.D. AUXILIARY HOSPITAL, LEPTON.—Miss Mary Gordon has been appointed matron. She was trained at Dumfries and Galloway Royal Infirmary and at Edinburgh Royal Infirmary. During the war she has served as a nursing sister of St. John Ambulance Brigade in France and England.

**NOTE.—Particulars of Appointments for insertion in this column will be welcomed.**

### THE COLLEGE OF NURSING

**(Limited by Guarantee).**

### FORTHCOMING LOCAL MEETINGS.

DERBY.—Thursday, June 27, 3.30 P.M., at the Guildhall, Derby, to establish a branch of the College of Nursing in Derby. The Mayor of Derby in the chair, supported by Admiral Sir Frederick Inglefield. Speakers, the Hon. Sir Arthur Stanley, M.P., G.B.E., C.B., and Miss Sparshott, R.R.C., Matron of the Royal Infirmary, Manchester.

BRISTOL.—Friday, July 5, 3 P.M., in the Museum Lecture Hall, University Road, to establish a branch of the College of Nursing in Bristol. The Lord Mayor will preside, supported by Sir Isambard Owen, F.R.C.P.

### Annual and Other Meetings.

### Paddington Green Children's Hospital.

AT the thirty-fifth annual meeting of subscribers to the Paddington Green Children's Hospital, Sir Douglas Owen, K.B.E., being in the chair, it was stated that a very large proportion of both in-patients and out-patients treated during 1917 were children of sailors and soldiers. The numbers of those cared for were given in THE HOSPITAL of June 22 (p. 267). Income last year was £10,036 and expenditure £7,054. Annual subscriptions maintained their level, being £997, as against £1,000 in 1916; but ordinary donations dropped to £1,275, £2,061 being the sum obtained a year previously. Mr. F. Stanley Cheer, of the East London Hospital for Children, has been appointed secretary of the institution, in succession to Mr. W. H. Pearce, of whom the committee speak as "the faithful friend and secretary of the institution during thirty-six years," and to whom a suitable pension has been granted.

### South London Hospital for Women.

THE Marchioness of Londonderry, chairman of the board of management, occupied the chair at the annual meeting of the South London Hospital for Women, held at 16 Carlton House Terrace by invitation of Viscountess Cowdray, one of the hon. treasurers. During the past year the number of in-patients treated was 976, and the average number of beds in daily occupation sixty-three. New out-patients cared for in 1917 numbered 7,095, and their total attendances 28,248. The board has received a gift of an excellent freehold site in the neighbourhood of Newington Causeway for the purpose of the extension of the out-patient department. It is estimated that at least £40,000 will be required to meet the cost of erection and equipment. Donations have increased during the period under review by more than £4,000, and a further sum of £20,000 has been presented to the hospital by anonymous donors for endowment purposes.

